# Equine Cemeteries

**Published:** 1882-01

**Authors:** 


Equine Cemeteries.—A curious feature in Japan is the decay of its temples, on the one hand, and the scrupulous care and attention paid to its burying places, on the other. In the outskirts of many villages, writes Miss Bird, in her "Unbeaten Tracks in Japan," there are cemeteries even for the horses. These are enclosures, situated near the human cemeteries. There are mounds of earth over each animal, and often a head-board. Considerable attention is paid to these places; the grass is kept neat, and flowers are placed on the graves. The Japanese horse is not a very fast or very amiable animal. Nor is it very well treated by its owners. Indeed, the Japs pay more respect to the horse dead than to the horse living.

